# Quality of life after stereotactic radiosurgery for brain metastasis: an assessment from a prospective national registry

**DOI:** 10.1007/s11060-024-04854-5

**Published:** 2024-10-21

**Authors:** Duy Q. Pham, Darrah E. Sheehan, Kimball A. Sheehan, Konstantinos Katsos, Camilo E. Fadul

**Affiliations:** 1 University of Virginia School of Medicine, Charlottesville, Virginia USA; 2 Department of Biomedical Engineering, University of Virginia Health System, Charlottesville, Virginia USA; 3 Division of Neuro-Oncology, Department of Neurology, University of Virginia Health System, Charlottesville, Virginia USA; 4 Department of Neurosurgery, Mayo Clinic, Rochester, Minnesota USA; 5 University of Virginia School of Medicine Inova Campus, 3300 Gallows Road, Falls Church, VA, 22042 USA

**Keywords:** Quality of life, Brain metastases, Depression, Pain, Stereotactic radiosurgery

## Abstract

**Purpose:**

Stereotactic radiosurgery (SRS) is frequently used in the management of brain metastasis patients. However, there is an urgent need to evaluate post-treatment outcomes and quality of life metrics for patients undergoing SRS for brain metastases.

**Methods:**

The NeuroPoint Alliance (NPA) SRS Quality Registry conducted prospective enrollment of patients undergoing SRS from 2017 to 2024. Patients with brain metastases from lung cancer, breast cancer, and melanoma were included in the analysis. Outcomes of interest included quality of life metrics, as captured by the five-dimension Euro-QOL (EQ-5D) at 6–12 months and last record follow-up, overall survival, local progression, out-of-field progression, and overall intracranial progression.

**Results:**

522 patients comprised our analytic cohort, and 315 patients had available EQ-5D data at the time of SRS and final follow-up. 264 (47.8%), 197 (35.7%), and 91 (16.5%) patients had 1, 2–4, and 5–14 lesions pre-SRS, respectively. The median overall survival time from diagnosis was 27.3 months. The median time-to-local progression was not reached. At final follow-up, 107 (34.0%) patients had improvement, 51 (16.2%) patients had stable, and 113 patients (35.9%) had worsening EQ-5D scores when compared to baseline. For 44 (13.9%) patients mixed responses across the EQ-5D indices were reported. Linear regression analysis showed that male sex, smoking status, primary tumor type, time-to-overall progression, cumulative intracranial tumor volume (CITV), and baseline EQ-5D were statistically significantly associated with EQ-5D single index at the final follow-up.

**Conclusion:**

Real-world data from the SRS NPA Registry demonstrated that most patients with brain metastasis had no change or improvement in quality of life after SRS. Baseline EQ-5D was predictive of EQ-5D single index at final follow-up, and, as such, EQ-5D at baseline would be a valuable assessment measure for brain metastasis patients undergoing SRS.

**Supplementary Information:**

The online version contains supplementary material available at 10.1007/s11060-024-04854-5.

## Introduction

Brain metastases are the most frequent intracranial tumors in adults, with an estimated incidence of 70,000-100,000 cases per year in the United States [1]. Non-small and small cell lung cancer, melanoma, breast, and renal cell carcinoma are among the most common primary tumors spreading to the central nervous system (CNS) [2]. With the increased use of brain MRI for screening, surveillance, and efficacy of cancer treatment modalities, the number of brain metastasis cases is expected to rise [3].

In addition to the usual stressors of cancer, patients with brain metastases expectedly carry heavy physical and psychological burdens due to potentially debilitating neurological symptoms [4, 5]. As such, increased effort has been placed into documenting patients’ health-related quality of life (QOL) indicators as a crucial outcome [6, 7]. Moreover, patients consistently cite QOL as one of the most important contributors to their treatment decisions [4, 8]. It is thus critical to have QOL prognostic information offered to patients with brain metastasis at the time of treatment decisions.

Stereotactic surgery (SRS) has become a mainstay in treating brain metastases as a minimally invasive procedure that allows for precise delivery of ionizing radiation to the intracranial sites of disease [3, 9]. SRS is more effective in achieving local tumor control and preserving cognitive function than whole-brain radiotherapy (WBRT) [9–11]. In terms of QOL, data have shown that SRS has positive effects on preserving patients’ pre-SRS functional capacity [3]. In this study, we aim to use real-world data from a national registry that prospectively compiled patient with brain metastases data from multiple centers to assess tumor control and QOL changes following SRS.

## Methods

### The NeuroPoint alliance quality registry for stereotactic radiosurgery

The NeuroPoint Alliance SRS Registry is a multi-institutional collaboration prospectively collecting information from patients undergoing SRS since 2017, with the most common pathologies treated being brain metastasis, meningiomas, and schwannomas [12]. The current database abstracts patient data from 27 active contributing sites with over 5500 patients enrolled and more than 8400 treatment events captured [13]. The registry was being used for quality improvement processes, and as such, was deemed IRB-exempt.

Data were gathered from eligible patients within the NPA registry who underwent SRS for the treatment of brain metastases originating from lung cancer, breast cancer, or melanoma and for whom there was at least one pre-SRS and one post-SRS QOL endpoint captured as part of their disease treatment progression.

### Baseline features, dosimetric characteristics & endpoints of interest

Baseline demographic data, functional information, disease characteristics, and treatment details of patients with brain metastases originating from lung cancer, breast cancer, and melanoma were included in the analysis. Baseline information included demographic information (e.g. age, sex, race); comorbidities and medical risk factors (e.g. diabetes mellitus, body mass index, coronary artery disease, smoking status); the Karnofsky Performance Status (KPS) score and patient-reported five-dimension Euro-QoL (EQ-5D-5 L in quality-adjusted life-years (QALYs – signifying how the patient’s quality of life compares to a full year in perfect health) as assessed by the clinical team [14, 15]. Disease characteristics included radiographic metrics (e.g. lesion maximum diameter, lesion volume, the cumulative intracranial tumor volume (CITV, defined as the sum of all intracranial lesions’ volumes)), number of brain metastases, the presence of intratumoral hemorrhage with or without extension into the brain parenchyma, and anatomic location of the lesions [16]. Treatment details consisted of dosimetry data (e.g. radiosurgical prescription dose, mean dose, margin dose, and number of fractions); pre-SRS treatments (e.g. surgical resection, whole-brain radiation therapy (WBRT), chemotherapy, immunotherapy, molecular therapy); the first metastatic site; and different neo-adjuvant treatments.

Outcomes of interest for the time-to-event analyses included all-cause mortality, local progression, out-of-field progression, and overall intracranial progression. Local progression was defined as an increase in the lesion volume by at least 72.8% compared to the baseline, corresponding to the volumetric equivalent of a 20% diameter increase of a spherical volume, by the Response Assessment in Neuro-Oncology Brain Metastasis (RANO-BM) criteria [17, 18]. Out-of-field progression was defined as the appearance of new metastasis beyond the previously irradiated field. Overall intracranial progression was a composite endpoint of the local and the out-of-field progression, considering the time point at which either local or out-of-field progression occurred first.

To assess QOL measures, we relied on the EQ-5D questionnaire responses. The EQ-5D is a self-reported patient survey designed to capture their description of five distinctive health states about their primary disease: mobility, self-care, usual activities, pain/discomfort, and anxiety/depression [19]. Patients are asked to indicate the level of problem they experience with each of the five dimensions, which collectively represent their EQ-5D profile. Subsequently, utilizing the *“eq5d”* package in RStudio, their profile data was converted to a single utility index value that ranges from 0, indicating death, to 1, indicating full health, using preference valuations weights for U.S. populations [19]. The SRS registry was queried for the baseline and follow-up EQ-5D questionnaire scores at 6–12 months and the last recorded follow-up, including the utility index scores given based on the patient’s responses for all five domains. Patients were included in the analytic sample with the availability of their SRS index scores pre-SRS and at the final follow-up. Regression analyses were then conducted to establish predictors of EQ-5D index and domain scores at final follow-up. We analyzed both the EQ-5D index and domain scores.

### Statistical analysis

Categorical variables were reported as proportions and frequencies, normally distributed continuous variables as means and standard deviations (SD), and non-parametric continuous variables as medians with interquartile ranges (IQR). Cut-off thresholds for continuous variables were determined based on tertile and quartile distributions to ensure adequate representation across the groups. These distributions were calculated for age, body mass index, CITV, margin dose, and mean dose. Following SRS guidelines, the lesion number at baseline was grouped as 1, 2–4, and ≥ 5 [20]. Missing baseline data were imputed using multivariate imputation by chained equations with 20 iterations and via the “*mice”* package [21]. Statistical significance was defined as a p-value < 0.05 level. Time-to-event analyses for overall survival, out-of-field, local, and intracranial progression were expressed by Kaplan-Meier curves. Multivariable Cox regressions were computed to establish hazard ratios for the associated risk factors for specific endpoints of interest. Statistical analyses were conducted using the *“survival”* and *“ggplot2”* in the R statistical package [22].

**Fig. 1:**
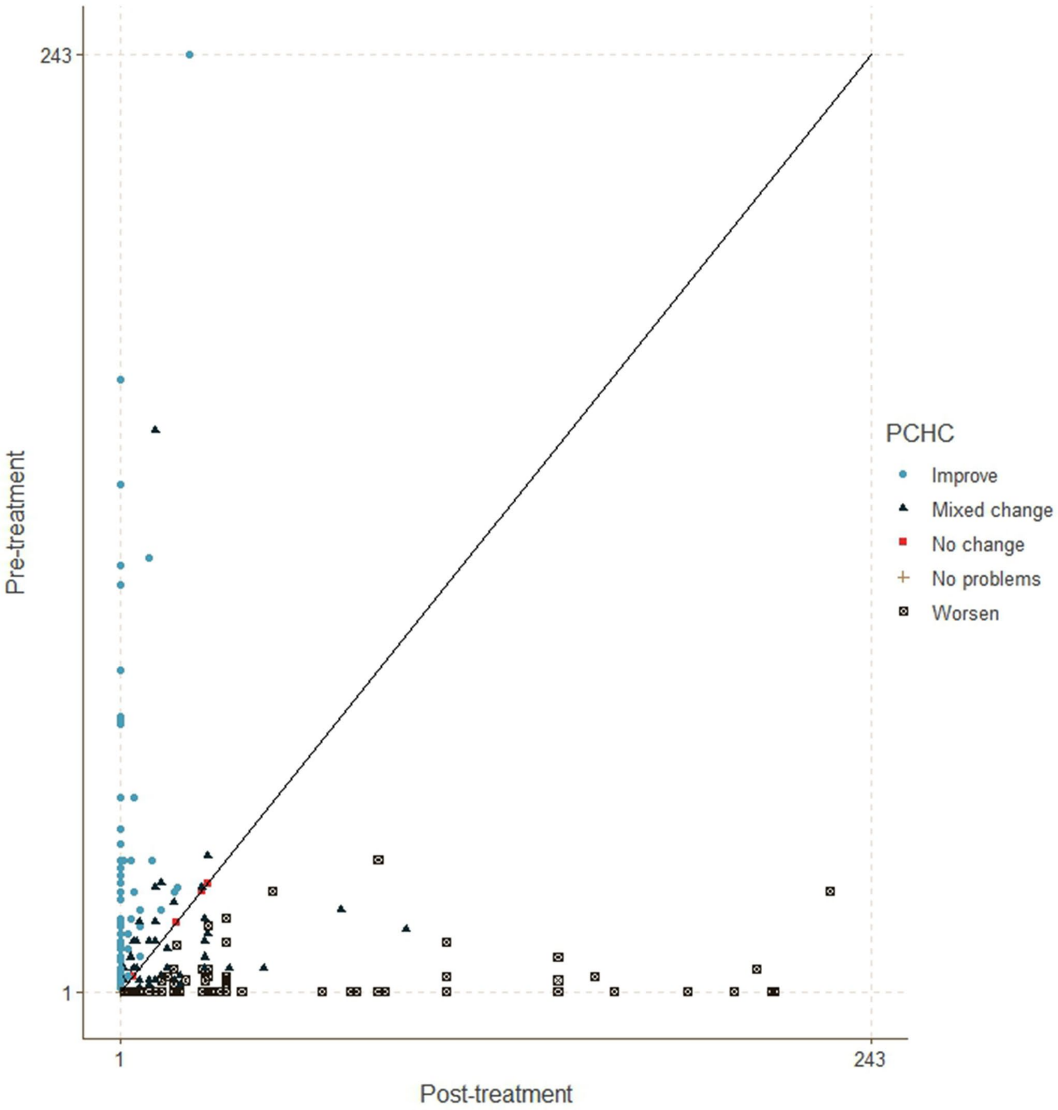
Health Profile Grid (HPG) depicting EQ-5D changes in individuals between baseline and final follow-up. The further a point is above the 45° line, the greater the improvement in an individual’s health. Conversely, the further below the line, the point is, the more their health has deteriorated. Those individuals on the line show “no change.”

The Paretian Classification of Health Change (PCHC) was utilized to analyze changes in patients’ EQ-5D scores over time [23]. PCHC categorizes changes in health status as better (improvement in at least one EQ-5D dimension), worse (deterioration in at least one dimension), mixed (both improvements and deteriorations in different dimensions), or unchanged. To visualize these changes, the Health Profile Grid (HPG) was used, which ranks health states from 1 (best) to 243 (worst) based on severity [23]. The HPG illustrates whether health has improved or worsened by plotting patients’ rankings at baseline and final follow-up: points above the 45° line indicate improvement, while points below indicate decline. The distance of a point from the 45° line reflects the magnitude of change, with patients on the line showing no change. Following the conversion of the health profiles to single indices, linear regression was conducted to identify predictors of EQ-5D at the final follow-up. In addition, the patients were categorized based on the direction of change of the EQ-5D single index. A paired t-test was conducted to compare pre-and post-SRS EQ-5D indices. Subsequently, logistic regression was performed to uncover predictors of EQ-5D change. These analyses were conducted using the “eq5d” and “stats” packages in R Fig. 1.

## Results

### Study cohort

Of the 3049 patients with brain metastasis in the NPA registry, 522 patients met the study inclusion criteria notes above. The patients originated from 15 participating sites from the SRS Quality Registry. Their median age at the time of SRS was 66.0 (IQR: 59.0–73.0) years, and 321 (58.2%) were female. The median follow-up was 8.8 months (IQR 3.4–18.5). The most common primary site was lung cancer (61.4%), followed by breast cancer (21.2%) and melanoma (17.4%). At the time of SRS, 264 (47.8%) patients had 1 intracranial lesion, 197 (35.7%) had 2–4 lesions, and 91 (16.5%) had more than 5 lesions. The primary tumor was controlled in 338 (61.2%) patients before SRS, with 140 (25.4%) undergoing systemic chemotherapy, and 71 (12.9%) receiving immunotherapy. The metastases were located in the frontal lobes in 319 (57.8%) patients, followed by the parietal lobe (37.9%), the temporal lobe (29.7%), and the occipital lobe (26.3%). The mean cumulative intracranial tumor volume was 4.0 (± 6.3) cm^3^. A summary of the cohort’s characteristics is presented in Table 1.

**Table 1:** Cohort characteristics

	Overall (*N* = 522)
Age, median (IQR)	66.0 (59.0–73.0)
Female sex, n (%)	321 (58.2%)
Race, n (%)	
Black	58 (10.5%)
White	443 (80.3%)
Other	51 (9.2%)
Body Mass Index, mean (SD)	26.4 (5.9)
Smoking, n (%)	269 (48.7%)
Diabetes mellitus, n (%)	87 (15.8%)
Coronary artery disease, n (%)	52 (9.4%)
Karnofsky Performance Status, n (%)	
100	170 (30.8%)
90	179 (32.4%)
80	115 (20.8%)
40–70	88 (15.9%)
EQ-5D score at baseline, mean (SD)	0.551 (0.443)
Primary Tumor Type, n (%)	
Breast	117 (21.2%)
Lung	339 (61.4%)
Skin	96 (17.4%)
Primary tumor controlled, n (%)	338 (61.2%)
Prior intralesional hemorrhage, n (%)	45 (8.2%)
Number of lesions at baseline, mean (SD)	2.9 (3.5)
Number of lesions at baseline, n (%)	
1	264 (47.8%)
2–4	197 (35.7%)
5–14	91 (16.5%)
Cumulative intracranial tumor volume, cm^3, mean (SD)	4.0 (6.3)
Cumulative intracranial tumor volume, cm^3, n (%)	
<0.56	184 (33.3%)
0.56–2.81	184 (33.3%)
>2.81	184 (33.3%)
Frontal lobe involvement, n (%)	319 (57.8%)
Parietal lobe involvement, n (%)	209 (37.9%)
Temporal lobe involvement, n (%)	164 (29.7%)
Occipital lobe involvement, n (%)	145 (26.3%)
Cerebellum involvement, n (%)	227 (41.1%)
Thalamus/basal ganglia involvement, n (%)	38 (6.9%)
Brainstem involvement, n (%)	36 (6.5%)
Pre-SRS systemic chemotherapy, n (%)	140 (25.4%)
Pre-SRS immunotherapy, n (%)	71 (12.9%)
Pre-SRS hormonal therapy, n (%)	12 (2.2%)
Pre-SRS WBRT, n (%)	46 (8.3%)
Pre-SRS molecular targeted therapy, n (%)	14 (2.5%)

### Overall survival

All-cause mortality was documented in 137 patients, with a median survival of 27.3 months. Supplemental Fig. 1A presents the Kaplan-Meier curve for overall survival. Multivariable Cox regression analysis revealed coronary artery disease, ≥ 5 lesions at the time of SRS, lack of primary tumor control, intralesional hemorrhage, and KPS ≤ 90 as independent risk factors for all-cause mortality. Supplemental Fig. 1B includes the hazard ratios and 95% confidence intervals of the Cox regression.

### Local, out-of-field, & intracranial progression

Local progression was recorded in 53 patients, and the median time-to-local progression was not reached. Multivariable Cox regression identified prior intralesional hemorrhage as an independent predictor of local progression.

Out-of-field progression was observed in 118 patients, with a median time-to-out-of-field progression corresponding to 14.9 months. More than 1 lesion at the time of SRS was predictive of out-of-field progression.

The composite outcome of overall intracranial progression was noted in 150 patients, and the median-to-overall intracranial progression was 14.0 months. More than 1 lesion at the time of SRS was predictive of overall intracranial progression, driven by out-of-field progression.

### EQ-5D at SRS and follow-up

Three-hundred-and-fifteen patients had available EQ-5D data at the time of SRS and at the final follow-up. The baseline EQ-5D index score at the time of SRS for the 522 patients was 0.551 (± 0.443). 107 (34.0%) patients indicated improvement from their EQ-5D baseline, while 51 (16.2%) were stable. One-hundred-thirteen patients (35.9%) had worsening scores, while 44 (13.9%) showed mixed responses across the EQ-5D indices (Table 2). One-hundred-and-sixty (50.8%) patients experienced no change in mobility, 66 (21.0%) improved, and 89 (28.3%) worsened. For the self-care domain, 232 (73.7%) patients did not report any change, 32 (10.2%) improved, and 51 (16.1%) worsened. Furthermore, 142 (45.1%) patients did not experience a change in their usual activities, 71 (22.5%) improved, and 102 (32.4%) deteriorated. For the pain/discomfort domain, 154 (48.9%) reported no change, 70 (22.2%) improved, and 91 (28.9%) worsened. Finally, 180 (57.1%) patients reported no change in anxiety/depression, 70 (22.2%) improved, and 65 (20.6%) worsened. Table 2 summarizes the overall and domain-specific changes for EQ-5D.

**Table 2:** Paretian Classification of Health Change for overall EQ-5D and EQ-5D domains at final Follow-up

		Number of Patients (*n*)	Percentage of Patients (%)
Overall	No Change	51	16.2
	Improve	107	34.0
	Worsen	113	35.9
	Mixed Change	44	13.9
Mobility	No Change	160	50.8
	Improve	66	21.0
	Worsen	89	28.3
Self-Care	No Change	232	73.7
	Improve	32	10.2
	Worsen	51	16.1
Usual Activities	No Change	142	45.1
	Improve	71	22.5
	Worsen	102	32.4
Pain/Discomfort	No Change	154	48.9
	Improve	70	22.2
	Worsen	91	28.9
Anxiety/Depression	No Change	180	57.1
	Improve	70	22.2
	Worsen	65	20.6

Linear regression showed that male sex, smoking status, primary tumor type, time-to-overall progression, CITV, and baseline EQ-5D were statistically significantly associated with EQ-5D single index at the final follow-up (Supplemental Table 1). Logistic regression identified smoking and CITV as negative predictors of EQ-5D change (Table 3). Overall survival probability was significantly higher in patients with stable/improved EQ-5D index scores relative to their worsening counterparts (Supplemental Fig. 2).

**Table 3:** Logistic regression for Eq. 5D change at final follow-up

	Odds Ratio (95% CI)	*p*-value
Patient Age	1.01 (0.98; 1.03)	0.72
Male	1.37 (0.73; 2.54)	0.32
Coronary Artery Disease	0.59 (0.21; 1.61)	0.30
Diabetes Mellitus	2.46 (0.93; 5.20)	0.18
Smoker	0.50 (0.27; 0.94)	0.03
Primary Tumor - Lung	1.55 (0.69; 3.47)	0.28
Primary Tumor - Skin	1.81 (0.65; 5.07)	0.25
CITV	0.93 (0.87; 0.99)	0.04
Primary Tumor Controlled	1.36 (0.75; 2.46)	0.26

### EQ-5D at 6–12 months follow-up

Ninety-three patients had EQ-5D data between 6- and 12 months following SRS, with a mean EQ-5D of 0.754 (± 0.217). There was a statistically significance change between baseline EQ-5D and 6- to 12-month EQ-5D (mean difference 0.03; *p* = 0.005) At 6- to 12-month follow-up, 28 (30.1%) patients indicated improvement from their EQ-5D baseline, while 17 (18.3%) were stable. Twenty-four (25.8%) had worsening responses, and another 24 (25.8%) showed mixed scores across the EQ-5D indices (Table 4). Fifty-five (59.1%) patients experienced no change in mobility, 19 (20.4%) improved, and 19 (20.4%) worsened. For the self-care domain, 67 (72.0%) patients did not report any change, 13 (14.0%) improved, and another 13 (14.0%) worsened. Furthermore, 50 (53.8%) patients did not experience a change in their usual activities, 21 (22.6%) improved, and 22 (23.6%) deteriorated. For the pain/discomfort domain, 53 (57.0%) reported no change, 19 (20.4%) improved, and 21 (22.6%) worsened. Finally, 50 (53.8%) patients reported no change in anxiety/depression, 22 (23.6%) improved, and 21 (22.6%) worsened. Table 3 summarizes the overall and domain-specific changes for EQ-5D at 6- to 12-month follow-up.

**Table 4:** Paretian classification of health change for overall EQ-5D and EQ-5D domains at 6–12 months follow-up

			Number of Patients (*n*)	Percentage of Patients (%)
**Overall**	**No Change**		17	18.3
	**Improve**		28	30.1
	**Worsen**		24	25.8
	**Mixed Change**		24	25.8
**Mobility**	**No Change**		55	59.1
	**Improve**		19	20.4
	**Worsen**		19	20.4
**Self-Care**	**No Change**		67	72.0
	**Improve**		13	14.0
	**Worsen**		13	14.0
**Usual Activities**	**No Change**		50	53.8
	**Improve**		21	22.6
	**Worsen**		22	23.6
**Pain/Discomfort**	**No Change**		53	57.0
	**Improve**		19	20.4
	**Worsen**		21	22.6
**Anxiety/Depression**	**No Change**		50	53.8
	**Improve**		22	23.6
	**Worsen**		21	22.6

Linear regression showed that coronary artery disease, smoking status, CITV, and baseline EQ-5D were statistically significantly associated with EQ-5D single index at 6- to 12-month follow-up (Supplemental Table 2). Logistic regression was not deemed feasible due to the smaller sample size of the subgroup analysis.

## Discussion

Historically, the most important outcome to determine the benefit of brain metastases treatment has been survival. QOL, however, has increasingly become a prominent outcome for patients who are surviving longer with advanced stages of cancer. Using real-world data from multiple institutions, our study aimed to expand on demonstrating the efficacy of SRS in preserving and potentially improving the patient-reported QOL after treatment for brain metastases. Our study, the largest to date to our knowledge, revealed patients with brain metastases following SRS had improvement (34.0%) or stability (16.2%) of their overall EQ-5D at the final follow-up. In addition, examining the same QOL indices at a shorter follow-up time of 6–12 months, a similar functional outcome was observed (18.3% with no change and 30.1% with improvement). We highlight at a minimum the preservation of baseline functional capacity in all five individual domains assessed by the EQ-5D questionnaire for both periods.

Our findings are consistent with the previously suggested benefit in QOL of SRS in patients with brain metastases related to preservation of cognitive function. In a single-center study, Bunevicius et al. showed that SRS preserved patients’ EQ-5D during their follow-up course [3]. In another study, Skeie et al. found that out of 97 patients enrolled, 66% showed improvement and 6% showed maintenance of their baseline QOL, assessed by the Functional Assessment of Cancer Therapy-Brain (FACT-BR), up to 12 months after their SRS procedure [24]. Other smaller studies using alternative questionnaires/indicators of QOL showed similar outcomes [25–29]. Our study builds upon these prior investigations by including a large multi-institutional cohort with real-world data suggesting that SRS provides a favorable therapeutic ratio when patient-reported QOL is the main outcome.

Although the extent of the benefit of SRS may vary according to the QOL domain, the relatively even spread of the positive outcomes of each EQ-5D domain showed the comprehensive benefits of SRS on different components of activities of daily living. However, the self-care domain notably showed a lower rate of improvement relative to the other four domains. One possible explanation is that the patients who are offered and ultimately opt for SRS for their metastases typically are not surgical candidates with significant disease burden and other medical comorbidities. The overall cancer treatment, including SRS, in such patients with considerable intracranial and extracranial disease burden can lead to fatigue and a still-difficult recovery process making self-care more challenging to perform.

It is important to note that anxiety/depression has been reported to be widely underdiagnosed for cancer patients as symptoms of the cancer as well as their treatments [30, 31]. Our findings of improvement in anxiety/depression in 22.2% of SRS-treated brain metastasis patients seem promising. They highlight the need for interdisciplinary collaboration- with psychiatrists, therapists, and social workers- to provide comprehensive care for these patients.

Cancer type appears to be associated with EQ-5D at follow-up. This is particularly true of lung cancer and melanoma which is positively associated with EQ-5D scores at the last follow-up. These findings concur with previous reports particularly of patients with brain metastases from lung cancer undergoing SRS [27, 32]. Although brain metastases can profoundly affect QOL, patient background, and pre-morbid factors are also important to consider in models examining treatment benefit. The effects of the two common comorbidities examined in our analyses, coronary artery diseases and diabetes, became blunted from the time of 6–12 months to the final follow-up. At the same time, both current smoker status and CITV remained significant negative predictors of QOL. These findings further suggest that the functional abilities of brain metastases patients depend in no small part on the degree of the intracranial burden. As metastases grow to affect more brain regions, it is expected the different eloquent pathways involved would create tangible neurological deficits impairing baseline functional abilities and associated QOL indices.

Our findings indicated a higher baseline EQ-5D associated with better QOL outcomes at the final follow-up. Moreover, overall survival was significantly higher in patients with stable or improved EQ-5D at final follow-up. As such, patients with low baseline EQ-5D can be a population where more attention is needed to identify patients for whom SRS will preserve or improve their functional capacities. It may be crucial to document EQ-5D scores at the time of presentation to provide patients and physicians with prognostic information about the effects of their disease course to aid in decision-making. Additionally, other quantitative measures such as the Recursive Partitioning Analysis (RPA), Updated-Recursive Partitioning Analysis (U-RPA), and Graded Prognostic Assessment (GPA) may be associated with QOL metrics but not presently included in the Registry [33]. The baseline documentation of these tools may allow studies that can determine their prognostic value to inform patients when making brain metastases therapeutic decisions.

Although the registry is prospective and the data is audited and curated, our study has several limitations inherent to the retrospective review. The accuracy of all data elements could not be independently verified at each site. The study suffered from significant attrition, which, although it reflects actual clinical practice, may have influenced our findings. While the data represent real-world patients, the practices from which the data are captured may not be fully generalizable to all sites that offer SRS because NPA registry participating sites are usually higher volume sites with tertiary referral patterns and considerable resources. The variability in clinical practices across sites resulted in inconsistent follow-up windows for some patients, complicating the comparison of EQ-5D outcomes post-SRS. Standardized protocols and a larger sample size would enhance the representation of diverse follow-up intervals, thereby improving the clinical relevance of comparisons. Additionally, the registry’s current limitations prevent the calculation of other prognostic indices like the RPA, U-RPA, and GPA. Moreover, there is currently a lack of information in regard to extracranial tumor control or progression that prevents our analysis into this presumptively contributory effect on QOL. This deficit is actively being improved by the team maintaining the NPA Registry with the hopes of improving future analyses. Lastly, EQ-5D only captures the progression of health-related QOL dimensions, and as constructed, it does not correlate well with cognitive function [34]. Despite these limitations, our study included a large cohort with baseline and follow-up patient-reported outcomes in several domains of QOL after SRS for brain metastases.

## Conclusion

Real-world data showed that SRS for brain metastases maintained or improved the quality of life, as determined by the EQ-5D scores, in most patients. Four domains of EQ-5D- anxiety/depression, usual activities, pain/discomfort, and mobility- had similar trends of improvement. The self-care domain was least likely to improve following SRS. Prior brain metastasis resection and baseline EQ-5D were significantly correlated with quality of life at the last follow-up. In addition to radiologic control and overall survival, patient-reported outcomes of value, like QOL, should be integrated into clinical practice for an informed shared decision of SRS for brain metastasis.

## Electronic Supplementary Material

Supplementary Material 1

## Data Availability

The data that support the findings of this study is not publicly available due to issues of sensitivity but is available from the corresponding author upon reasonable request.

## References

[1] Nayak L, Lee EQ, Wen PY (2012) Epidemiology of brain metastases. Curr Oncol Rep 14:48–54 22012633 10.1007/s11912-011-0203-y

[2] Cagney DN, Martin AM, Catalano PJ et al (2017) Incidence and prognosis of patients with brain metastases at diagnosis of systemic malignancy: a population-based study. Neuro-Oncol 19:1511–1521 28444227 10.1093/neuonc/nox077 PMC5737512

[3] Bunevicius A, Lavezzo K, Shabo L et al (2020) Quality-of-life trajectories after stereotactic radiosurgery for brain metastases. J Neurosurg 134:1791–1799 32650308 10.3171/2020.4.JNS20788

[4] Verhaak E, Gehring K, Hanssens PEJ et al (2019) Health-related quality of life of patients with brain metastases selected for stereotactic radiosurgery. J Neurooncol 143:537–546 31073966 10.1007/s11060-019-03186-z PMC6591192

[5] Lassman AB, DeAngelis LM (2003) Brain metastases. Neurol Clin 21:1–23 12690643 10.1016/s0733-8619(02)00035-x

[6] Bunevicius A, Donovan L, Sheehan J (2022) Health-related quality of life trajectories after stereotactic radiosurgery for brain metastases: a systematic review. J Neurooncol 159:319–331 35788469 10.1007/s11060-022-04067-8

[7] Bottomley A, Pe M, Sloan J et al (2016) Analyzing data from patient-reported outcome and quality of life endpoints for cancer clinical trials: a start in setting international standards. Lancet Oncol 17:e510–e514 27769798 10.1016/S1470-2045(16)30510-1

[8] Zeng KL, Raman S, Sahgal A et al (2017) Patient preference for stereotactic radiosurgery plus or minus whole brain radiotherapy for the treatment of brain metastases. Ann Palliat Med 6:S155–S160 28866900 10.21037/apm.2017.06.11

[9] Michalopoulos GD, Katsos K, Grills IS et al (2023) Stereotactic radiosurgery in the management of non–small cell lung cancer brain metastases: a prospective study using the NeuroPoint Alliance Stereotactic Radiosurgery Registry. J Neurosurg 1:1–10 10.3171/2023.8.JNS23308 37948684

[10] Sheehan JP, Grills I, Chiang VL et al (2018) Quality of life outcomes for brain metastasis patients treated with stereotactic radiosurgery: pre-procedural predictive factors from a prospective national registry. J Neurosurg 131:1848–1854 30579284 10.3171/2018.8.JNS181599

[11] Chang EL, Wefel JS, Hess KR et al (2009) Neurocognition in patients with brain metastases treated with radiosurgery or radiosurgery plus whole-brain irradiation: a randomised controlled trial. Lancet Oncol 10:1037–1044 19801201 10.1016/S1470-2045(09)70263-3

[12] Sheehan JP, Michalopoulos GD, Katsos K et al (2024) The NeuroPoint alliance SRS & tumor QOD registries. J Neurooncol 166:257–264 38236549 10.1007/s11060-023-04553-7

[13] AANS Stereotactic Radiosurgery Registry (2024) Neuropoint, https://www.neuropoint.org/stereotactic-radiosurgery-registry/ (accessed 13

[14] EuroQol Group (1990) EuroQol–a new facility for the measurement of health-related quality of life. Health Policy Amst Neth 16:199–208 10.1016/0168-8510(90)90421-9 10109801

[15] Schag CC, Heinrich RL, Ganz PA (1984) Karnofsky performance status revisited: reliability, validity, and guidelines. J Clin Oncol off J Am Soc Clin Oncol 2:187–193 10.1200/JCO.1984.2.3.187 6699671

[16] Hirshman BR, Wilson BR, Ali MA et al (2018) Cumulative intracranial tumor volume augments the Prognostic Value of Diagnosis-Specific Graded Prognostic Assessment Model for Survival in patients with Melanoma Cerebral Metastases. Neurosurgery 83:237–244 28973506 10.1093/neuros/nyx380

[17] Lin NU, Lee EQ, Aoyama H et al (2015) Response assessment criteria for brain metastases: proposal from the RANO group. Lancet Oncol 16:e270–278 26065612 10.1016/S1470-2045(15)70057-4

[18] Oft D, Schmidt MA, Weissmann T et al (2021) Volumetric regression in Brain metastases after Stereotactic Radiotherapy: Time Course, Predictors, and significance. Front Oncol 10:590980 33489888 10.3389/fonc.2020.590980 PMC7820888

[19] Devlin PDN, Parkin D, Janssen B (2020) An Introduction to EQ-5D Instruments and Their Applications. In: Methods for Analysing and Reporting EQ-5D Data. Springer, Cham. 10.1007/978-3-030-47622-9_1 33347096

[20] Gondi V, Bauman G, Bradfield L et al (2022) Radiation Therapy for Brain metastases: an ASTRO Clinical Practice Guideline. Pract Radiat Oncol 12:265–282 35534352 10.1016/j.prro.2022.02.003

[21] van Buuren S, Groothuis-Oudshoorn K (2011) Mice: multivariate imputation by chained equations in R. J Stat Softw 45:1–67

[22] R: The R Project for Statistical Computing, https://www.r-project.org/ (accessed 16 (2024)

[23] Devlin NJ, Parkin D, Browne J (2010) Patient-reported outcome measures in the NHS: new methods for analysing and reporting EQ-5D data. Health Econ 19:886–905 20623685 10.1002/hec.1608

[24] Skeie BS, Eide GE, Flatebø M et al (2017) Quality of life is maintained using Gamma Knife radiosurgery: a prospective study of a brain metastases patient cohort. J Neurosurg 126:708–725 27058206 10.3171/2015.10.JNS15801

[25] Verhaak E, Schimmel WCM, Gehring K et al (2021) Health-related quality of life after Gamma Knife radiosurgery in patients with 1–10 brain metastases. J Cancer Res Clin Oncol 147:1157–1167 33025282 10.1007/s00432-020-03400-w PMC7954744

[26] Vesagas TS, Aguilar JA, Mercado ER et al (2002) Gamma knife radiosurgery and brain metastases: local control, survival, and quality of life. J Neurosurg 97:507–510 12507086 10.3171/jns.2002.97.supplement

[27] Bragstad S, Flatebø M, Natvig GK et al (2017) Predictors of quality of life and survival following Gamma Knife surgery for lung cancer brain metastases: a prospective study. J Neurosurg 129:71–83 28820304 10.3171/2017.2.JNS161659

[28] Randolph DM, McTyre E, Klepin H et al (2017) Impact of radiosurgical management of geriatric patients with brain metastases: clinical and quality of life outcomes. J Radiosurgery SBRT 5:35–42 PMC5675506 29296461

[29] Miller JA, Kotecha R, Barnett GH et al (2017) Quality of life following stereotactic radiosurgery for single and multiple brain metastases. Neurosurgery 81:147–155 28327994 10.1093/neuros/nyw166

[30] Passik SD, Dugan W, McDonald MV et al (1998) Oncologists’ recognition of depression in their patients with cancer. J Clin Oncol 16:1594–1600 9552071 10.1200/JCO.1998.16.4.1594

[31] Adjei Boakye E, Osazuwa-Peters N, Mohammed KA et al (2020) Prevalence and factors associated with diagnosed depression among hospitalized cancer patients with metastatic disease. Soc Psychiatry Psychiatr Epidemiol 55:15–23 31444517 10.1007/s00127-019-01763-1

[32] Gerosa M, Nicolato A, Foroni R et al (2005) Analysis of long-term outcomes and prognostic factors in patients with non—small cell lung cancer brain metastases treated by gamma knife radiosurgery. J Neurosurg 102:75–80 15662785 10.3171/jns.2005.102.s_supplement.0075

[33] Fadul CE, Sarai G, Bovi JA et al (2023) Relevance of the updated recursive partitioning analysis (U-RPA) classification in the Contemporary Care of patients with brain metastases. Cancers 15:3255 37370865 10.3390/cancers15123255 PMC10297002

[34] Ophuis RH, Janssen MF, Bonsel GJ et al (2019) Health-related quality of life in injury patients: the added value of extending the EQ-5D-3L with a cognitive dimension. Qual Life Res 28:1941–1949 30887386 10.1007/s11136-019-02156-2 PMC6571080

