# Supplementary material for: Quality of life after stereotactic radiosurgery for brain metastasis: an assessment from a prospective national registry
Source: J Neurooncol. 2024 Oct 21;171(2):383–91. doi: 10.1007/s11060-024-04854-5 (PMC11695642; doi:10.1007/s11060-024-04854-5)

**Supplemental Table 1:** Linear regression for EQ5D at final follow-up.

|  | **Coefficient (95% CI)** | **p-value** |
| --- | --- | --- |
| Patient Age | 1.01 (0.99; 1.03) | 0.52 |
| **Male** | **0.07 (0.01; 0.12)** | **0.02** |
| Coronary Artery Disease | -0.08 (-0.17; 0.01) | 0.08 |
| Diabetes Mellitus | 0.02 (-0.04; 0.09) | 0.48 |
| **Smoker** | **-0.08 (-0.14; 0.03)** | **<0.001** |
| **Primary Tumor - Lung** | **0.08 (0.01; 0.15)** | **0.02** |
| **Primary Tumor - Skin** | **0.10 (0.01; 0.19)** | **0.04** |
| **Time to Overall Progression** | **0.02 (0.01; 0.03)** | **0.04** |
| **CITV** | **-0.01 (-0.01; 0.00)** | **<0.001** |
| **EQ5D - Baseline** | **0.57 (0.40; 0.73)** | **<0.001** |

**Supplemental Table 2:** Linear regression for EQ5D at 6-12 months follow-up.

|  | **Coefficient** | **p-value** |
| --- | --- | --- |
| Patient Age | 0.01 (-0.01; 0.05) | 0.43 |
| Male | 0.08 (-0.01; 0.17) | 0.09 |
| **Coronary Artery Disease** | **-0.16 (-0.29; -0.03)** | **0.02** |
| Diabetes Mellitus | 0.01 (-0.08; 0.11) | 0.78 |
| **Smoker** | **-0.1 (-0.19; -0.01)** | **0.03** |
| Primary Tumor - Lung | 0.09 (-0.03; 0.21) | 0.14 |
| Primary Tumor - Skin | 0.09 (-0.07; 0.24) | 0.27 |
| Time to Overall Progression | 0.01 (-0.01; 0.02) | 0.39 |
| **CITV** | **-0.01 (-0.02; -0.01)** | **0.01** |
| **EQ5D - Baseline** | **0.59 (0.40; 0.73)** | **<0.001** |

**Supplemental Figure 1A:** Kaplan Meier curve for overall survival for patients with brain metastases.
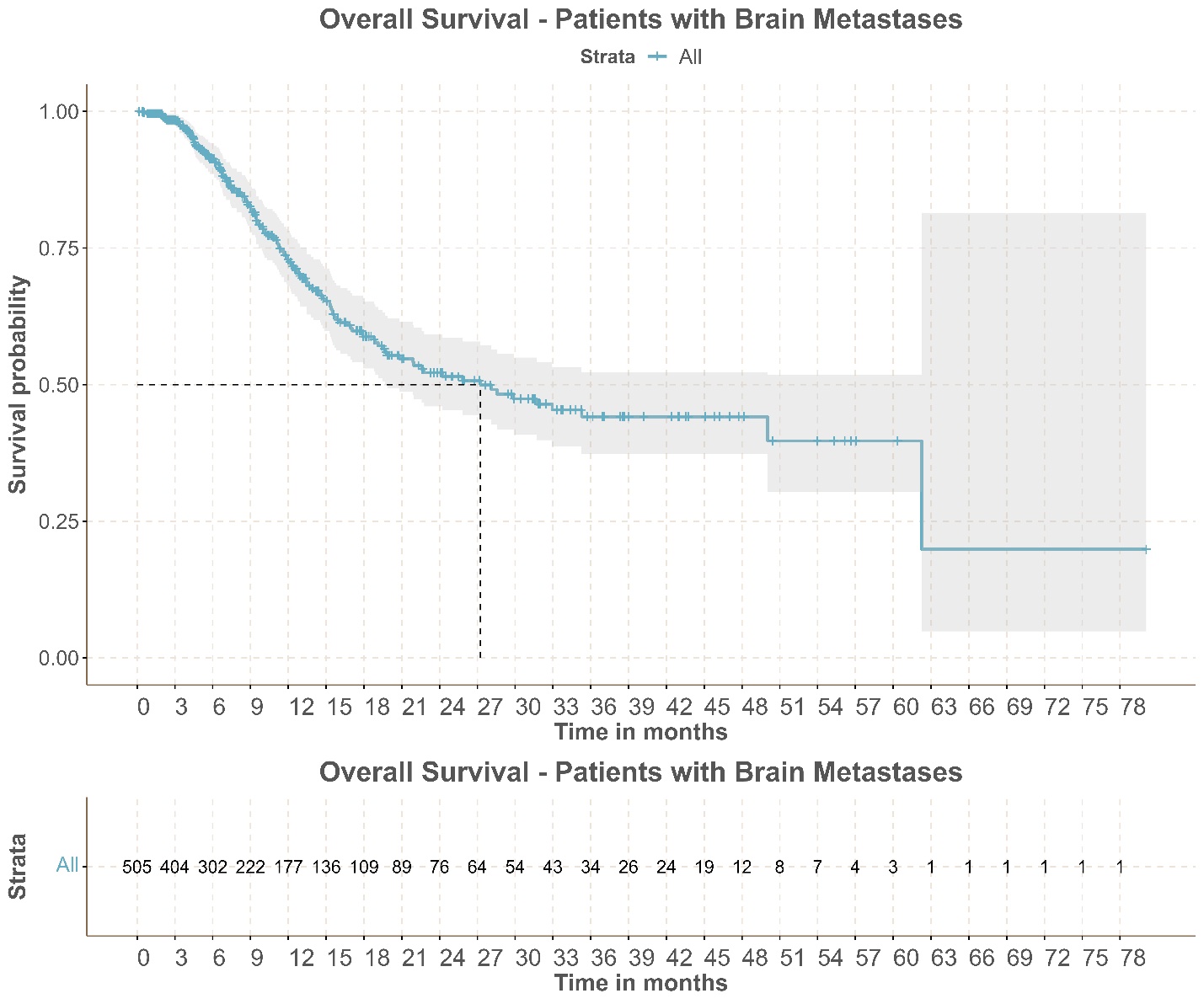


**Supplemental Figure 1B:** Cox regression for overall survival


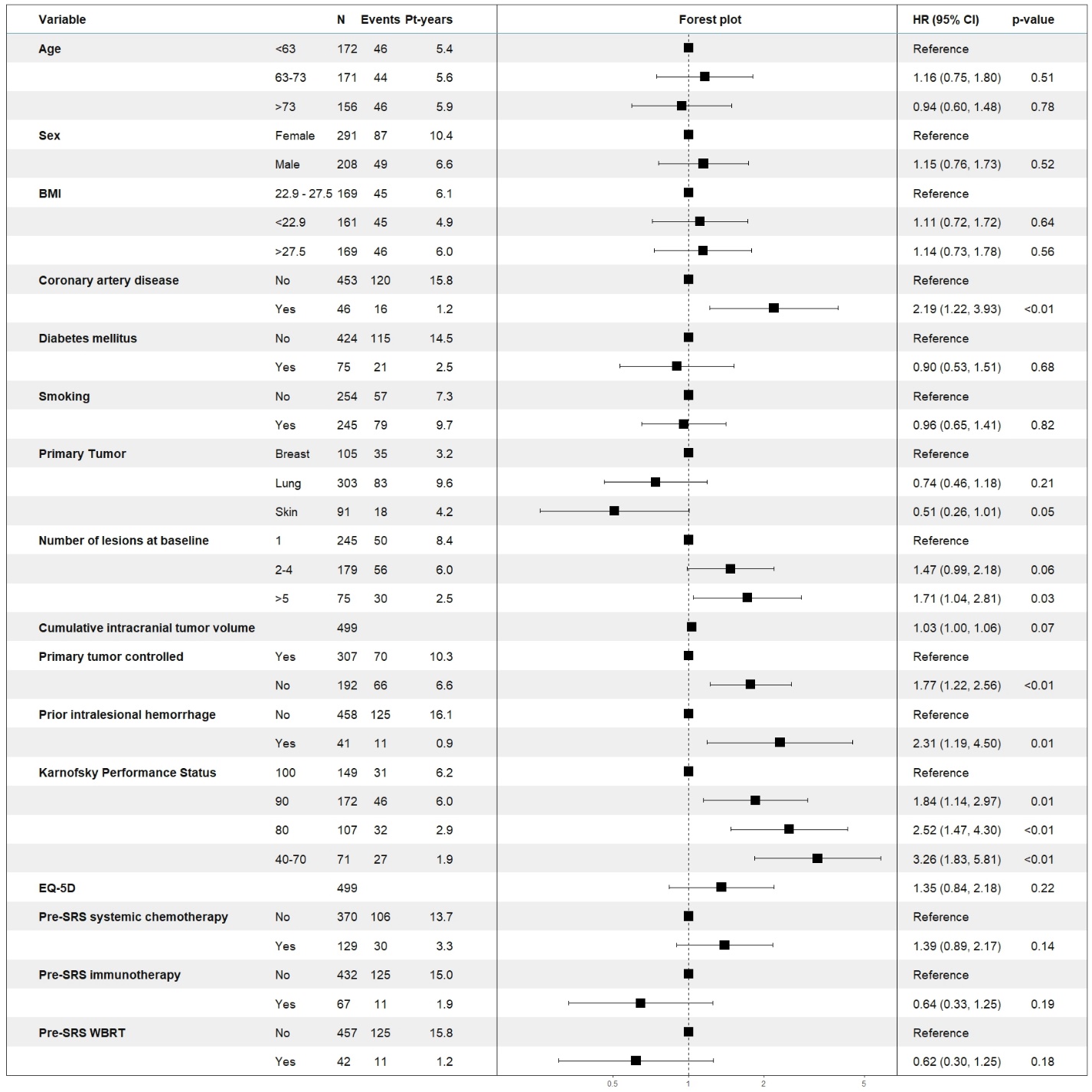


**Supplemental Figure 2.** Kaplan-Meier survival curves comparison for patients with stable/improved EQ-5D and patients with worsened EQ-5D.

Of the 315 patients who have available EQ-5D information at the time of their final follow-up, only 214 patients have recorded outcomes data. The remaining 101 patients were not included in this survival analysis.


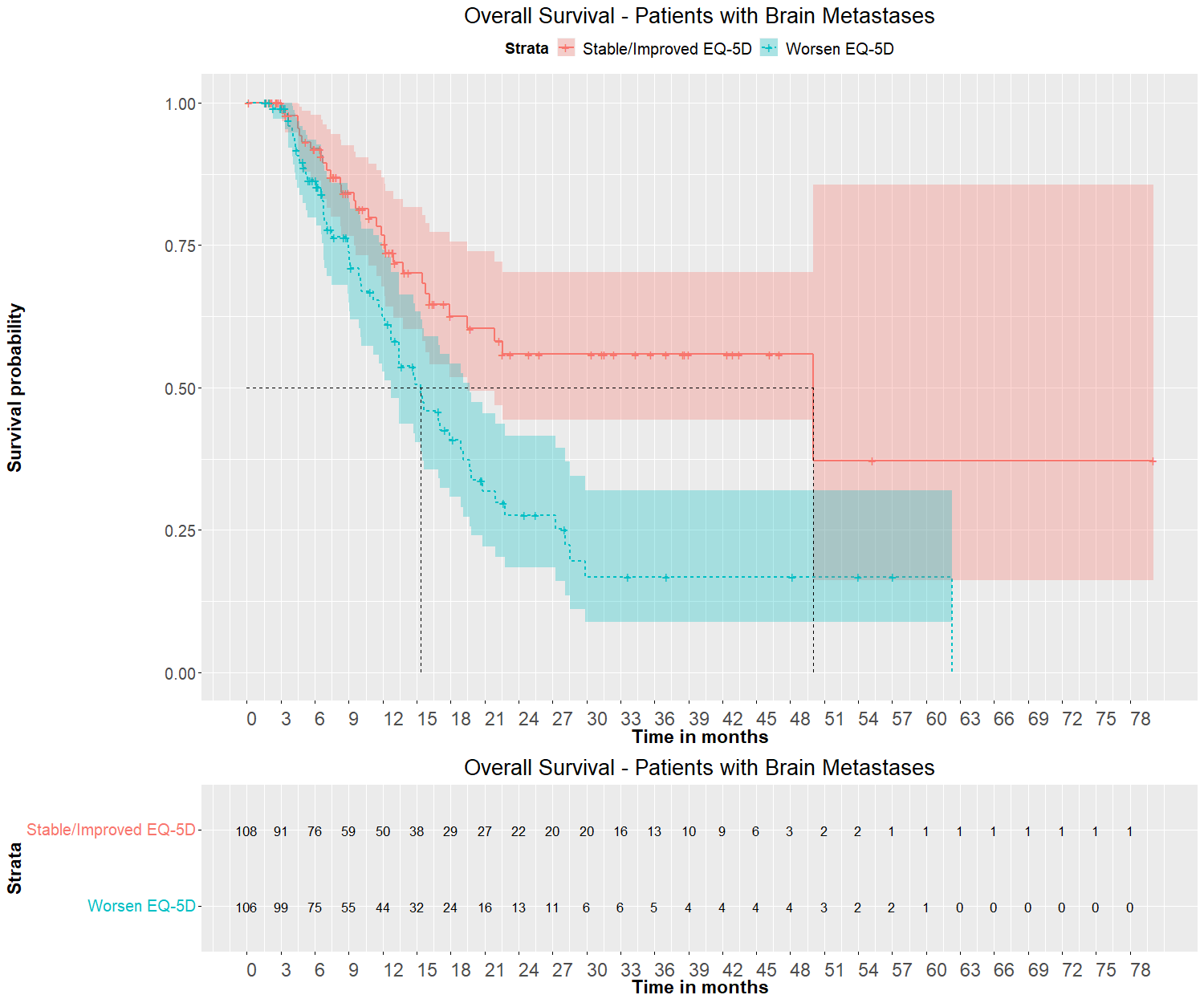

Supplement: Supplementary file 1 — Supplementary Material 1 [file 11060_2024_4854_MOESM1_ESM.docx]
